# Multiple Myeloma Presenting as Acute Renal Failure in the Absence of Other Characteristic Features

**DOI:** 10.7759/cureus.1703

**Published:** 2017-09-20

**Authors:** Zachary N Gastelum, Diana M Biggs, Aaron Scott

**Affiliations:** 1 University of Arizona College of Medicine-Tucson; 2 Hematology and Oncology, University of Arizona Cancer Center

**Keywords:** multiple myeloma, acute renal failure, acute kidney injury, plasma cell dyscrasia, bence jones, light chain

## Abstract

This case report describes a 54-year-old, asymptomatic man who presented with hyperkalemia on routine lab testing who was later found to have acute renal failure, unresponsive to fluid resuscitation, with minimal improvement after hemodialysis. After a comprehensive evaluation ruled out common causes of acute renal failure, the patient underwent testing with a bone survey, urine protein electrophoresis (UPEP), serum protein electrophoresis (SPEP), and immunoelectrophoresis for suspected plasma cell dyscrasia and received plasmapheresis for hyperviscosity syndrome and nephrotoxicity, which resulted in improved renal function. Lab results showed monoclonal gammopathy, elevated serum free light chains, and Bence Jones protein in the urine with a follow-up bone marrow biopsy indicating plasma cell dyscrasia. The patient received a diagnosis of multiple myeloma (MM) and was started on chemotherapy and immunosuppression. In patients presenting with acute renal failure with an evaluation ruling out prerenal and postrenal causes, multiple myeloma should be considered.

## Introduction

Multiple myeloma (MM) is a plasma cell malignancy defined by a serum monoclonal spike (M-spike) > 3 g/dL or more than 10% clonal plasma cells in the bone marrow along with one myeloma-defining event which includes the following: anemia, hypercalcemia, renal insufficiency, or bone lesions [1]. Multiple myeloma often presents with acute or chronic renal failure. Interpretation of renal disease in patients with multiple myeloma may be complicated by additional causes of renal failure such as nonsteroidal anti-inflammatory drugs (NSAIDs), infection, and dehydration [2]. While renal impairment is a frequent presenting symptom, it rarely is the singular presenting symptom of multiple myeloma, as a majority of patients also present with hypercalcemia, anemia, and/or pubic bone lesions [3]. In this case, we present a patient with acute renal failure (ARF) as his only manifestation of a plasma cell dyscrasia without additional classic features, illustrating the importance of considering multiple myeloma as a viable etiology for acute kidney injury even in the absence of other symptoms and signs characteristic of multiple myeloma, or in the presence of other common causes of acute renal failure.

## Case presentation

A 54-year-old male with type 2 diabetes mellitus, diagnosed one year prior, was admitted for acute renal failure. His active medications included metformin, insulin glargine, lisinopril, and atorvastatin. The patient underwent routine laboratory blood tests one day prior to presentation, which were significant for a potassium of 8 mEq/L for which he was advised to go to the emergency department where he had a creatinine (Cr) of 11.7 mg/dL. The patient was transferred to a regional hospital where repeat laboratory tests showed an estimated glomerular filtration rate (eGFR) of 5 mL/min, a blood urea nitrogen (BUN) of 134 mg/dL, creatinine of 11.7 mg/dL (with a baseline Cr of 1.46 mg/dL, three months prior), potassium of 6.2 mEq/L, and metabolic acidosis. The patient reported taking 800 mg of ibuprofen, nightly, for three days for muscle cramps. The patient was otherwise asymptomatic and had no other significant past medical history. Family history and the social history were non-contributory. Physical examination was notable for hypertension but was otherwise unremarkable.

Laboratory values on admission were notable for a hemoglobin of 13.7 gm/dL, potassium of 6.2 mEq/L, bicarbonate of 15 mEq/L, creatinine of 11.7 mg/dL, BUN of 134 mg/dL, phosphorus of 8.3 mg/dL, calcium 9.6 mg/dL, and uric acid of 11.5 mg/dL. Urinalysis was positive for proteinuria and a urine sodium of 76 mEq/L. These lab values, in addition to the patient’s volume status, and vital signs were important in distinguishing pre-renal causes from intrinsic renal causes. Baseline creatinine was obtained from patient’s primary care physician which showed a creatinine of 1.46 mg/dL from three months prior, consistent with ongoing renal dysfunction before hospitalization. Given extreme elevation in creatinine within the span of three months time, the patient was diagnosed with acute renal failure.

The patient was volume resuscitated with intravenous fluids for decreased renal perfusion, secondary to dehydration. Lisinopril and metformin home medications were held due to concern for worsening acute renal failure and acidosis. The patient underwent extensive laboratory and imaging studies after there was no response to fluid resuscitation. The comprehensive laboratory panel included: hepatitis B surface antigen (HBsAg), hepatitis C antibody (HCV Ab), anti-nuclear antibody (ANA), anti-neutrophil cytoplasmic antibody (ANCA), and glomerular basement membrane antibody (GBM Ab) which were all negative. Renal ultrasound was performed which showed a right kidney measuring 12.9 x 6.5 x 7.4 cm and a left kidney measuring 11.4 x 6.1 x 6.2 cm. Renal size was normal, and there was no evidence of renal mass lesions, hydronephrosis, or calcifications. Nephrology and Vascular Surgery were consulted for further management.

A radiographic bone survey revealed a focal, nonspecific, lucent lesion within the right frontal bone, but no focal lytic lesions in the central axial skeleton or proximal appendicular skeleton were present. Serum immunoglobulin analysis revealed the following: IgA 35 mg/dL, IgG 978 mg/dL, and IgM <5 mg/dL. Peripheral blood smear showed no abnormalities. Serum protein electrophoresis (SPEP) was positive for a monoclonal spike (M-spike) of 1.56 g/dL in the gamma region and the urine protein electrophoresis (UPEP) showed 148 mg/dL of protein consistent with lambda light chains. Serum immunoelectrophoresis showed kappa light chains and lambda light chains at 2.87 mg/dL and 801 mg/dL, respectively, and a kappa: lambda light chain ratio of <0.01. Upon abnormal SPEP and UPEP results, hematology and oncology were consulted for suspected MM. He was treated with plasmapheresis for five days for hyperviscosity syndrome and nephrotoxicity related to lambda light chain disease. This intervention resulted in improvement of the patient’s renal function, as evidenced by a decrease in creatinine to 3.3 mg/dL at the time of discharge.

Subsequent bone marrow biopsy and aspirate with clot were performed along with flow cytometry indicating a 3.1% monoclonal lambda plasma cell population. The patient was also started on a chemotherapy regimen of dexamethasone 40 mg and bortezomib 3.25 mg prior to discharge for management of MM. The patient was discharged home with weekly dexamethasone and bortezomib, and was instructed to follow-up with his outpatient oncologist for ongoing management of his MM.

## Discussion

Multiple myeloma, a malignant monoclonal proliferation of plasma cells, commonly presents with hypercalcemia, renal failure, anemia, and bone lesions (the CRAB features), which are used in the diagnostic evaluation for MM. This constellation of symptoms consist of hypercalcemia and bone pain secondary to lytic bone lesions and increased osteoclastic activity, with these symptoms evident in 28% and 58% of MM patients on presentation, respectively. Anemia is the most common manifestation of MM, found in about 73% of MM patients at presentation [4]. Non-CRAB features such as infection and hyperviscosity of the blood may also be present in MM patients, though they are not included in the diagnostic criteria of MM. Renal failure is the focus of this case report and occurs most commonly from pathologic light chain deposition in the kidneys. Elevated creatinine is found in about 48% of MM patients at presentation [4]. This case report illustrates acute renal failure as the initial and sole presentation of MM in the absence of the other signs and symptoms that comprise the CRAB features. Isolated acute renal failure as an initial presentation of multiple myeloma has previously been reported; however, the vast majority of such cases were accompanied by hypercalcemia and/or anemia (≤12 g/dL), which was not seen in this patient.

Renal dysfunction is present in most MM patients, and the etiology of renal dysfunction in MM includes various mechanisms including paraprotein or non-paraprotein associated renal complications. The ongoing renal failure in MM often results from tubular nephropathy because of circulating paraproteins secreted by plasma cell clones, most commonly immunoglobulins and free light chains [5].

A previous analysis of multiple myeloma patients showed that renal dysfunction prior to diagnosis most frequently manifested as acute renal failure, followed by chronic renal failure, and lastly, nephrotic syndrome. This study found that 84% of the 50 MM patients presented with renal impairment before a diagnosis of MM. This same study determined that acute renal failure was the most common manifestation of renal dysfunction in MM [6]. In another study from the Oxford Regional Renal Unit of 56 patients with multiple myeloma, renal failure was the initial presentation of MM in 50% of patients; however, a great majority also presented with hypercalcemia as a potential precipitant of renal failure [7]. Though there is substantial evidence that renal failure is a common presentation of MM, there is less evidence suggesting renal failure as a sole presenting entity in patients with MM.

A 2011 study published by the Medical Oncology Journal addresses the notion of renal disease as a presenting or preceding feature of MM. In this case, a patient with renal dysfunction apparent on laboratory results was found to have light chain deposition and findings consistent with membranoproliferative glomerulonephritis in the absence of immune complex deposition. At the time she presented with renal dysfunction, no signs or positive diagnostic tests for MM were identified. However, 28 months later, the patient met diagnostic criteria for multiple myeloma [8]. This case report emphasizes that renal dysfunction, as evidenced by proteinuria and increased serum creatinine, may be the initial presentation of MM. Furthermore, it is important to consider the eventual development of MM in patients with renal failure; thus, it may be beneficial to recommend MM testing follow-up in certain patients. Another study analyzed 26 MM patients who were referred for acute renal failure, 88% of which had no diagnosis of MM at time of referral. The only other presenting symptom in 100% of the patients was anemia. This study concluded that MM should be considered as a cause of unexplained acute renal failure in patients older than 50 [9]. These conclusions set a good foundation for our case study and allow us to take a step further to suggest considering a diagnosis of MM in acute renal dysfunction patients in the absence of anemia, hypercalcemia and bone pain/lytic bone lesions.

Upon initial laboratory testing, history, and physical exam, the differential diagnosis for this case included chronic renal failure, most likely secondary to diabetic nephropathy or acute renal failure, of which there are many possible causes. Acute to subacute onset etiologies were most likely found in our patient given his baseline creatinine of 1.49 mg/dL, three months prior, and normal-sized kidneys on renal ultrasound.

In determining the cause of the patient’s ARF, it was important to distinguish whether his ARF was prerenal, intrinsic renal, or postrenal. Given patient’s euvolemic status, stable vital signs, BUN/creatinine ratio <20:1 mg/dL, and urine sodium of 79 mEq/L, prerenal causes were less likely. Furthermore, the absence of obstruction or hydronephrosis on renal ultrasound suggested that a postrenal cause was not the source of this patient’s ARF. Thus, it was concluded that the patient’s ARF was secondary to an intrinsic renal pathologic process. Given that the patient was normocalcemic upon presentation (corrected calcium 9.4 mg/dL), was experiencing no bone pain, and had a hemoglobin of 13.7 gm/dL, multiple myeloma was quite low on the differential diagnosis. Despite this, the SPEP and UPEP were ordered.

Considering the patient’s history, the following differential diagnoses were first considered: ARF secondary to recent NSAID use; however, this would have been an unlikely sole cause of the patient’s acute renal failure, given a duration of only three days of standard dose NSAID use. Dehydration was on the initial differential, however, this was less likely due to euvolemia and urine electrolytes. Diabetic nephropathy was considered given the patient’s proteinuria, however, this was less likely due to his lack of retinopathy and neuropathy. Other diagnoses that were considered included tubular injury from recent unknown antibiotic use (however, acute tubular necrosis (ATN) secondary to medication was unlikely given lack of casts on urinalysis) and rhabdomyolysis in the setting of recent leg cramps, secondary to statin use (patient had started atorvastatin two months prior to presentation); however, the patient’s creatine kinase was within normal limits. Of the other causes of acute intrinsic renal failure, tumor lysis syndrome, hemolysis, thrombotic thrombocytopenic purpua and hemolytic-uremic syndrome (TTP/HUS), contrast-induced nephropathy, and atheroembolism were all unlikely causes given the lack of risk factors and pertinent negative history of previous chemotherapy, surgery, medications, or infection.

Although the patient lacked bone pain, pathologic fractures, weakness, severe anemia (hemoglobin <10 gm/dL), infection (often pneumococcal), hypercalcemia (serum calcium level >10 mg/dL), and obvious lytic bone lesions on bone survey, MM was considered as a cause of this patient’s intrinsic renal failure. Thus, UPEP, SPEP, and immunoelectrophoresis tests were ordered. They returned positive for an IgG lambda light chain monoclonal gammopathy and Bence Jones proteinuria with MM suspected by plasma cell dyscrasia on bone marrow biopsy and aspirate. Though the patient’s bone marrow aspirate does not meet diagnostic criteria for MM (defined as a bone marrow plasma cell population (BMPC) >10%), 3-5% of MM patients will have a BMPC <10% and require repeat bone marrow biopsy, computed tomography (CT) or magnetic resonance imaging (MRI) for confirmation of BMPC >10% or plasmacytoma [10]. Furthermore, evidence of end-organ damage attributable to light chain deposition ruled out the diagnosis of monoclonal gammopathy of undetermined significance (MGUS). It was concluded that the patient’s ARF was secondary to light chain nephropathy associated with MM, possibly worsened by recent NSAID use.

Oftentimes, the diagnosis of MM may not be considered in patients with renal insufficiency due to the absence of the other characteristic signs and symptoms of that disease. However, this case and the other studies addressed in this report emphasize the importance of recognizing all possible causes of renal failure, including MM, even in the absence of other typical features. To the best of our knowledge, acute renal failure as an initial presentation of MM in the absence of anemia, hypercalcemia, and lytic bone lesions has rarely been reported.

## Conclusions

Renal manifestations of MM consist of an array of renal pathology including both acute and chronic renal failure, proteinuria, and nephrotic syndrome. MM is an important consideration as the cause of a patient presenting with acute renal failure of unknown etiology, specifically in the absence of prerenal or postrenal etiology. Characteristic features of MM including hypercalcemia, anemia, bone pain, lytic bone lesions, and recurrent infection may be absent on presentation, and renal insufficiency may be the only sign present. NSAID use, dehydration, and infection, which are typical causes of acute renal failure in and of themselves, may merely be precipitants of renal disease secondary to multiple myeloma.
